# Quantitative Detection of µ Opioid Receptor: Western Blot Analyses Using µ Opioid Receptor Knockout Mice

**DOI:** 10.2174/157015911795016921

**Published:** 2011-03

**Authors:** Shinya Kasai, Hideko Yamamoto, Etsuko Kamegaya, George R Uhl, Ichiro Sora, Masahiko Watanabe, Kazutaka Ikeda

**Affiliations:** a Division of Psychobiology, Tokyo Institute of Psychiatry, Tokyo 156-8585, Japan; b Molecular Neurobiology Branch, National Institute on Drug Abuse, National Institutes of Health, Bethesda, Maryland 21224, USA; c Department of Neuroscience, Division of Psychobiology, Tohoku University Graduate School of Medicine, 980-8574 Sendai, Japan; d Department of Anatomy and Embryology, Hokkaido University Graduate School of Medicine, Sapporo, 060-8638, Japan

**Keywords:** Knockout mice, µ opioid receptor, quantification, Western blot analysis.

## Abstract

Increasing evidence suggests that µ opioid receptor (MOP) expression is altered during the development of and withdrawal from substance dependence. Although anti-MOP antibodies have been hypothesized to be useful for estimating MOP expression levels, inconsistent MOP molecular weights (MWs) have been reported in studies using anti-MOP antibodies. In the present study, we generated a new anti-MOP antibody (N38) against the 1-38 amino acid sequence of the mouse MOP *N*-terminus and conducted Western blot analysis with wildtype and MOP knockout brain lysates to determine the MWs of intrinsic MOP. The N38 antibody detected migrating bands with relative MWs of 60-67 kDa in the plasma membrane fraction isolated from wildtype brain, but not from the MOP knockout brain. These migrating bands exhibited semi-linear density in the range of 3-30 µg membrane proteins/lane. The N38 antibody may be useful for quantitatively detecting MOP.

## INTRODUCTION

The µ opioid receptor (MOP) belongs to the superfamily of seven-transmembrane-spanning G protein-coupled receptors. Many pharmacological data using gene knockout mice have shown that MOP is a preferred target of opioid drugs, such as morphine and fentanyl, and that MOP appears to play a critical role in mediating major clinical effects of opioids, including analgesia, dependence, tolerance, and respiratory depression [1].

MOP expression has been reportedly altered in substance abuse [2]. MOP protein expression decreases after ethanol treatment in various brain regions [3]. MOP binding potential also decreases in the cortex and increases in the striatum, and altered expression is associated with alcohol craving in alcohol-dependent subjects undergoing alcohol withdrawal [4, 5]. Furthermore, during chronic cocaine treatment and withdrawal from chronic cocaine administration, MOP mRNA levels increase in the nucleus accumbens [6, 7]. However, in other brain regions such as limbic regions, chronic cocaine treatment downregulates MOP mRNA expression [8].

MOP has been detected by numerous methods, including Northern blot analysis, RNase protection assay, real-time reverse transcription polymerase chain reaction (RT-PCR), immunohistochemistry, and Western blot analysis. Among these detection methods, Western blot analysis with MOP-specific antibodies is effective for quantitatively measuring MOP protein expression. Although a number of anti-MOP antibodies have been generated, these anti-MOP antibodies produce immunoreactive bands with a diverse range of molecular weights (MWs). MOP MWs are different between glycosylated and non-glycosylated types [9, 10]. Additionally, MOP forms dimers whose MWs are different from monomers [9]. MOP MWs also differ as a result of various isolating conditions, such as pH and the presence or absence of reducing agents [11].

In the present study, we generated a new anti-MOP antibody against the 1-38 amino acid sequence of the mouse MOP amino-terminus (*N*-terminus) and tested it with other anti-MOP antibodies generated against different MOP regions using brain lysates from MOP knockout mice. Furthermore, we investigated the linearity of MOP detection using the N38 antibody in the physiological range of protein concentrations.

## MATERIALS AND METHODS

### Animals

C57BL/6J mice were purchased from CLEA Japan Inc. (Tokyo, Japan). MOP knockout mice were generated as previously described [12]. The targeted allele of the MOP gene lacks exon 1, which encodes the coding region corresponding to 95 amino acids of the MOP *N*-terminus starting with methionine. After backcrossing to C57BL/6J for 10 generations, MOP knockout mice were maintained to cross between heterozygotes to produce all genotype littermates. MOP knockout mouse genotyping was performed by PCR using GoTaq DNA polymerase (Promega, Madison, WI, USA) with a common forward primer and two reverse primers at the *neo*-resistant gene only in the knockout allele and at the 5’ flanking region of the MOP gene only in the wildtype allele. The mice were housed five per cage in an environment maintained at 22 ± 2°C with 55 ± 5% relative humidity and a 12 h light/12 h dark cycle (lights on 8:00 a.m. to 8:00 p.m.). The mice had *ad libitum *access to a standard laboratory diet and water. The experimental procedures were approved by the Institutional Animal Care and Use Committee of the Tokyo Institute of Psychiatry.

### Antibody Production

The nucleotide sequences encoding the *N*-terminus of mouse MOP (amino acid residues 1-38, Accession No. U19380) were amplified by PCR using a single-strand cDNA library prepared from the adult mouse brain and sub-cloned into the Bam*HI*/Eco*RI* site of the pGEX4T-2 plasmid vector (Amersham Biosciences, Bucks, UK) to be expressed as the glutathione *S*-transferase (GST) fusion protein. The fusion protein was emulsified with Freund’s complete adjuvant in the first immunization and incomplete adjuvant in the subsequent immunization (DIFCO, Detroit, MI, USA) and injected subcutaneously into female rabbit at intervals of 2 weeks. Two weeks after the sixth injection, the MOP-specific antibody (N38) against the 1-38 amino acid sequences of the *N*-terminus of mouse MOP was collected by affinity purification using GST-free polypeptides coupled to CNBr-activated Sepharose 4B (Amersham Biosciences). GST-free peptides were prepared by in-column thrombin digestion of GST fusion proteins bound to glutathione-Sepharose 4B media.

### Membrane Preparation from Brain

The cortex from adult male mouse brain was collected and homogenized in ice-cold 0.32 M sucrose solution containing 1 mM ethylene glycol tetraacetic acid (EGTA) and protease inhibitors (Roche, Basel, Switzerland). The total homogenate (T) was subjected to centrifugation at 800 × *g* for 12 min at 4ºC to remove the nuclei (P1), and the supernatant was further centrifuged at high speed at 22,000 × *g* for 20 min at 4ºC. The supernatant (S2) was removed, and the pellet (P2) was resuspended in 0.32 M sucrose solution containing 1 mM EGTA and protease inhibitor and used as the membrane fraction for Western blot analyses. Protein content was determined by the Bradford method using a Bio-Rad protein assay kit (Bio-Rad Laboratories, Hercules, CA, USA) according to the manufacturer’s instructions.

### Western Blot Analyses

The anti-MOP antibodies 44-308G and AB5511 against chemically synthetic peptides from the internal region and carboxy-terminus (*C*-terminus), respectively, of MOP were purchased from Biosource International Inc. (Camarillo, CA, USA) and Chemicon International Inc. (Hampshire, UK), respectively. Protein samples were diluted in Laemmli’s sample buffer, and 0.3-30 µg of total protein/lane was subjected to sodium dodecyl sulfate-polyacrylamide gel electrophoresis (SDS-PAGE) using 5-20% gradient SDS-polyacrylamide gels (ATTO, Tokyo, Japan). The resolved proteins were transferred onto polyvinylidene difluoride (PVDF) membranes (Nippon Genetics Co. Ltd., Tokyo, Japan) using a Trans-Blot SD transfer cell (Bio-Rad). Following transfer, membranes were washed with Tris-buffered saline containing 0.05% Tween-20 (TBST) and then blocked in Blocking One (Nacalai Tesque Inc., Kyoto, Japan) for 1 h at room temperature. The membranes were then incubated for 5 days with N38 (1:100-1:400) or for 1 day with 44-308G (1:5,000) or AB5511 (1:5,000-1:20,000) rabbit polyclonal antibodies in TBST containing 10% Blocking One at 4ºC. After washing three times for 10 min in TBST, membranes were incubated with peroxidase-labeled secondary antibody (1:50,000; Zymed Laboratories Inc., South San Francisco, CA, USA) for 1 h at room temperature. The membranes were washed in TBST, incubated with Super-Signal WestDura Extended Duration Substrate (Pierce Biotechnology Inc., Rockford, IL, USA) for 10 min, and exposed to electrogenerated chemiluminescence (ECL) films (Amersham Biosciences). ECL films were scanned densitometrically, and the optical density of bands was quantified using Image J version 1.38 software (http://rsb.info.nih.gov/ij/; accessed October 9, 2009).

## RESULTS

Anti-MOP antibodies have been produced since the early 1990s and have yielded inconsistent results with regard to the MW of the MOP protein. We generated a new antibody against the 38 amino acid sequence of the mouse MOP *N*-terminus (MDSSAGPGNISDCSDPLAPASCSPAPGS-WLNLSHVDGN) and determined the antibodies’ immunological specificities for MOP by Western blot analyses.

First, the N38 anti-MOP antibody was compared with the other antibodies (44-308G and AB5511) which were commercially available. The 44-308G and AB5511 antibodies were generated against the internal region of human MOP and the *C*-terminus of rat MOP, respectively. Plasma membrane proteins (P2 fraction) derived from the cortex of adult male C57BL/6J mice were resolved by SDS-PAGE, transferred onto PVDF membrane, and stained with three anti-MOP antibodies. Numerous bands around 30-110 kDa were stained in the total homogenate (T) with all three antibodies (Fig. (**1**)). Approximately 40, 44, 60-67, and 95 kDa proteins were stained with the N38 antibody for 5 days (indicated by arrowheads in Fig. (**1**) left panel). However, no immunoreactive bands were detected with the N38 antibody for 1 day (data not shown). 30, 47-55, 55, 60, and 95 kDa proteins were detected with the 44-308G antibody, and 33-38, 40-45, 50, and 60-67 kDa proteins were detected with the AB5511 antibody for 1 day (indicated by arrowheads in center and right panels in Fig. (**1**)). In these immunoreactive bands, only the migrating bands with relative MWs of 60-67 kDa stained by either N38 or AB5511 antibodies were accumulated in the P2 fraction (indicated by arrows in Fig. (**1**)).

For identification of MOP in a number of bands detected by three anti-MOP antibodies, Western blot analyses were performed with the P2 fraction prepared from the cortex of MOP knockout mice previously generated by Sora *et al.* (1997) [12]. These MOP knockout mice lacked exon 1, including the first methionine of mouse MOP, and exhibited virtually no MOP immunoreactivity in the spinal cord dorsal horn [12]. The 60-67 kDa migrating bands were completely abolished in the knockout P2 fraction stained with N38 or AB5511 antibodies (indicated by arrows in left and right panels in Fig. (**2**)). The intensity of two weak bands around 40-45 kDa was attenuated in the knockout P2 fraction (indicated by arrowhead in Fig. (**2**)). The other bands that did not accumulate in the C57BL/6J P2 fraction were detected in the knockout P2 fraction with three anti-MOP antibodies. These results indicate that the N38 and AB5511 antibodies detected intrinsic MOP proteins as 60-67 kDa migrating bands in the P2 fraction from adult brain.

We additionally conducted Western blot analyses with high antibody concentrations to detect quantitatively smaller amounts of MOP, such as physiological levels of MOP protein expression. The N38 and AB5511 antibodies were used at a dilution of 1:100 and 1:5,000, respectively. The migrating bands of MOP proteins were detected at concentrations of 1-30 μg and 3-30 μg total protein/lane by N38 and AB5511 antibodies in the P2 fraction from adult male C57BL/6J brain, respectively (indicated by arrows in Fig. (**3A**)). The density of migrating MOP protein bands exhibited semi-linearity at 3-30 μg total protein/lane in Western blot analyses with either N38 or AB5511 antibodies (Fig. (**3B**)). More intense MOP bands were detected by N38 than by AB5511 antibodies, suggesting that the N38 antibody is more suitable than AB5511 for quantitative assay of MOP protein levels by Western blot analysis.

## DISCUSSION

The N38 anti-MOP antibody increased 60-67 kDa migrating bands in the wildtype P2 fraction but not in the MOP-knockout P2 fraction. In a similar range of MWs, the same patterns of migrating bands were detected as MOP only by Arvidsson *et al.* (1995) [13] and Chalecka-Franaszek *et al.* (2000) [14]. Similar to other G protein-coupled receptors, MOP contains sites for *N*-linked glycosylation (Asn-X-Cys/Ser/Thr) in the extracellular *N*-termini in the mouse, rat, and human MOP (four positions in mouse MOP and five positions in rat and human MOP). The MWs of MOP treated with various glycosidases were markedly reduced to a range of 40-50 kDa [9, 10], suggesting that glycosyl residues greatly contributed to MOP MWs. The variations of glycosylation, such as the type and number of glycosylated residues, may result in migrating bands of MOP. The two weak bands around 40-45 kDa in the wildtype P2 fraction were virtually eliminated in the MOP knockout P2 fraction by staining with AB5511 antibody (right panel in Fig. (**2**)). Unknown is whether non-glycosylated MOP exists in physiological conditions or derives from deglycosylation during isolation of the P2 samples, but these two weak bands may correspond to non-glycosylated MOP.

The N38 anti-MOP antibody is against the 1-38 amino acid sequence of mouse MOP that exists in the extracellular *N*-terminus domain of MOP. The *N*-terminus of the MOP protein is more diversified than other regions in mouse, rat, and human (21 amino acid differences in the first 100 amino acids of the *N*-terminus, two differences in the next 200 amino acids, and five differences in the last 100 amino acids). Additionally, both *N*- and *C*-termini of mouse opioid receptors show a greater variety of amino acid sequences than the internal region among the three types of mouse opioid receptors. The relatively unconserved sequence of the MOP *N*-terminus may cause the high reactivity and specificity of the N38 anti-MOP antibody. Although a control experiment should be conducted with MOP-absorbed N38 antibody because of remaining nonspecific immunoreactivity, this antibody may be useful for studies detecting MOP proteins in mouse tissues.

## Figures and Tables

**Fig. (1):**
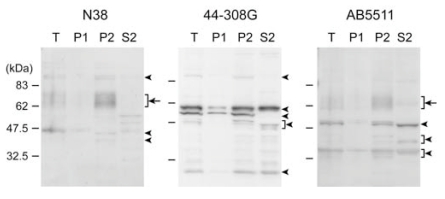
Western blot analyses with three anti-MOP antibodies produced against peptides from different MOP regions. Anti-MOP rabbit polyclonal antibodies (N38, 44-308G, and AB5511) were produced against the *N*-terminus, internal region, and *C*-terminus of MOP, respectively. Total cortex homogenate (T) from adult male C57BL/6J mice was fractionated to nuclear fraction (P1), cytoplasmic membrane fraction (P2), and cytoplasm fraction (S2) by two-step centrifugation at 800 × *g* and subsequently 22,000 × *g*. 10 µg of each fraction was subjected to SDS-PAGE. Protein blots were incubated with anti-MOP antibodies at appropriate dilution (N38, 1:400; 44-308G, 1:5,000; AB5511, 1:20,000), followed by reaction with secondary antibody and visualization.

**Fig. (2):**
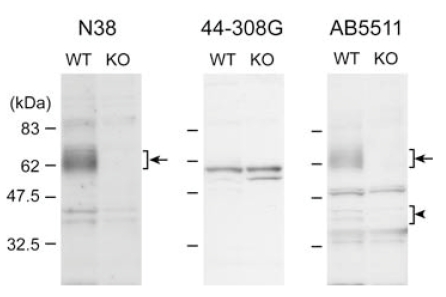
Western blot analyses at the P2 fraction derived from wildtype and *Oprm1* knockout mice. The P2 fractions were isolated from the cortex of wildtype (WT) and *Oprm1* knockout (KO) adult male mice. 10 µg of the P2 fraction from each genotype was subjected to SDS-PAGE. Protein blots were reacted with anti-MOP antibodies at appropriate dilution (N38, 1:400; 44-308G, 1:5,000; AB5511, 1:20,000), followed by reaction with secondary antibody and visualization.

**Fig. (3):**
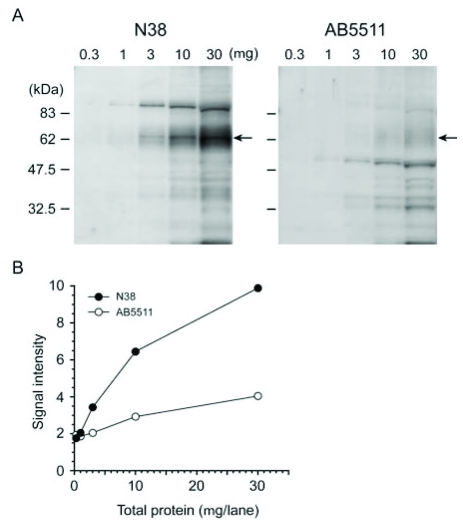
Western blot analyses with N38 and AB5511 antibodies for MOP detection in a protein dose-dependent manner. The P2 fractions were isolated from the cortex of adult male C57BL/6J mice. 0.3, 1, 3, 10, and 30 µg of proteins were subjected to SDS-PAGE. Protein blots were reacted with N38 (1:100) and AB5511 (1:5,000) antibodies, followed by reaction with secondary antibody and visualization (**A**). The optical density of migrating bands indicated by arrows around 60-67 kDa was quantified using Image J software (**B**).
